# Hazards&Robots: A dataset for visual anomaly detection in robotics

**DOI:** 10.1016/j.dib.2023.109264

**Published:** 2023-05-24

**Authors:** Dario Mantegazza, Alind Xhyra, Luca M. Gambardella, Alessandro Giusti, Jérôme Guzzi

**Affiliations:** IDSIA - Dalle Molle Institute for Artificial Intelligence USI-SUPSI, Polo universitario Lugano - Campus Est, Via la Santa 1, CH-6962 Lugano-Viganello

**Keywords:** Out of distribution detection, Intelligent robotics, Deep learning, Computer vision

## Abstract

We propose Hazards&Robots, a dataset for Visual Anomaly Detection in Robotics. The dataset is composed of 324,408 RGB frames, and corresponding feature vectors; it contains 145,470 normal frames and 178,938 anomalous ones categorized in 20 different anomaly classes. The dataset can be used to train and test current and novel visual anomaly detection methods such as those based on deep learning vision models. The data is recorded with a DJI Robomaster S1 front facing camera. The ground robot, controlled by a human operator, traverses university corridors. Considered anomalies include presence of humans, unexpected objects on the floor, defects to the robot. Preliminary versions of the dataset are used in [1,3]. This version is available at [12]

**Specifications Table**

**Table:** 

Subject	Artificial Intelligence, Computer Vision and Pattern Recognition
Specific subject area	Dataset for developing and testing Visual Anomaly Detection systems for Autonomous Robots
Type of data	Images, Annotations per sample in CSV files Feature Embeddings from CLIP ViT-B/32 [2]
How the data were acquired	We record the feed of a ground robot DJI Robomaster front camera on a microSD or via the Robomaster app. The robot is teleoperated in more than 550 sorties, in 6 different corridors located on two different floors of a university campus. In all recordings the robot moves at the center, along the length of the corridor. The recordings vary in duration, location, presence of anomalies, illumination, and corridor traversing direction; this is done to cover more scenarios. The recordings are divided in frames, resized and features are extracted.
Data format	Raw Analyzed Encoded
Description of data collection	In the videos the robot fully traverses random sections of the corridor. In normal videos no anomalies are present. In anomalous ones we manually place anomalies; anomalies are always visible and identifiable. In short video, the anomalies are at 2m from a start position; the robot moves towards the anomaly while keeping it in frame, then it stops at ∼5cm and moves back.
Data source location	Institution: USI-SUPSI university building "East Campus" City: Lugano Country: Switzerland Latitude and Longitude: 46.01237, 8.96139
Data accessibility	Repository name: Zenodo Data identification number: https://doi.org/10.5281/zenodo.7859211 Direct URL to data: https://zenodo.org/record/7859211

## Value of the Data

• This is a dataset for visual anomaly detection for robotics. The dataset is recorded in a real environment. The dataset differs from existing anomaly detection datasets [4,5] as those are usually collected in fixed, controlled settings; ours is collected by a mobile robot with environmental variables such as reflections, changes in illumination or variations in the surroundings.
• Thanks to its large number of frames, the dataset provided can be used to train and test Deep Learning models for the task of Visual Anomaly Detection.
• The data can be useful for researchers working on both applied and theoretical research. It can be used by applied researchers to replicate real robot issues and test their solutions or by theoretical researchers to benchmark their models on a realistic anomaly detection task.
• The dataset is composed of 324,408 512 × 512 images and the same number of 512-sized feature vectors. All samples are labeled, we provide a suggested splits into training (only normal), training (mixed), validation and test sets.
• The dataset provides both images and pre-extracted features embeddings to allow a larger variety of approaches to the task of Anomaly Detection.

## 1 Objective

The state-of-the-art models and approaches [6], [7], [8], [9], [10] for Anomaly detection in images are often trained and tested on datasets that strongly differ from what is encountered by autonomous robots [4,^5^,8]. Thus, we collected a dataset to act as a proxy of realistic robotic settings making it possible to develop and benchmark anomaly detection systems for robots that use cameras.

## 2 Data Description

The dataset is composed of 8 files:

- *README.md* the readme files containing some basic information about the dataset,
- *embeddings.zip* a zip file containing 324,408 512-sized features vectors extracted using a CLIP ViT-B/32 from the images. The vectors are divided in 4 PyTorch .pt files for fast loading,
- *metadata.zip* a zip file containing the annotation files for the dataset*,*
- *training_set.zip* a compressed folder containing 47,157 video frames,
- *training_mixed_set.zip* a compressed folder containing 144,603 video frames,
- *validation_set.zip* a compressed folder containing 2,801 video frames,
- *test_set.zip* a compressed folder containing 129,846 video frames,
- *code.zip* a compressed folder containing the code used in the frames creation, for reading the dataset and additional custom PyTorch dataset Classes for both the embeddings and images.

Regardless of the representation in frames or embeddings, the data is split in four disjoint sets:

- *Training,* containing only normal samples,
- *Training_mixed,* containing both normal and anomalous samples,
- *Validation,* containing only normal samples,
- *Testing,* containing normal and anomalous samples.

A summary of the four sets is in Table 1.

**Table 1:** The dataset's split summary.

Set	Total Samples	Anomalous Samples
Training (normal)	47,157	0
Training (mixed)	144,603	94,362
Validation	2802	0
Test	129,846	84,576

The compressed folders (*<set_name>_set.zip*) contain JPEG images with a separate sequential numeration for each folder. The file naming follows this style: 000000_512_512.jpg is the 1^st^ sample of the set (the file numeration starts at 0) and 001234_512_512.jpg is the 1235^th^ sample in the set.

In *embeddings.zip,* we provide four PyTorch files and four Numpy files; each file represents one of the sets. The naming convention is <set_name>_embs.pt for PyTorch files and <set_name>_embs.npy for Numpy ones. When loaded with the dataset classes provided in the *code.zip*, the result is a PyTorch tensor of shape (set_size, 512); for example, for the validation set the shape of the tensor in validation_embs.pt is (2802, 512). Be sure to use the correct code provided for Numpy or PyTorch embeddings. These vectors correspond to embeddings of the image encoder component of the CLIP model ViT-B/32 publicly provided by Open AI [2].

In the *metadata.zip* compressed folder we provide 6 CSV files and 2 TXT files.

Two CSV files contain the frame labelling; each CSV file provides two columns; *frame_id* with the sample ids and *label* with the label id of the sample.

- test_frames_labels.csv,
- training_mixed_frames_labels.csv,

The other four CSV files contain the corridor labelling for each sample of a set; each CSV provides a *frame_id* column and a *env* column; the latter indicates the corridor id of a sample.

- training_set_frames_envs.csv,
- training_mixed_set_frames_envs.csv,
- validation_set_frames_envs.csv,
- test_set_frames_envs.csv,

The two TXT files contain the mapping between ids and names of labels and environments:

- labels_mapping.txt,
- envs_mapping.txt,

The environments are 3: *long, short,* and *underground.* We did this as the corridors can be grouped in these three classes.

*Long* corridors have a wooden wall, *short* corridors are larger and have chairs and tables, and *underground* corridors are concrete corridors with maintenance tubing and cabling on the ceiling.

In the dataset we provide 21 different classes, in Table 2 we list all labels and their ids.

**Table 2:** Classes of Anomalies in the dataset.

Id	Name	Number of samples in Training	Number of samples in Testing
0	Normal	47,157 (training) +50,241 (training mixed) +2802 (validation) =100,200	45,270
1	Box	5868	4976
2	Cable	5460	5201
3	Cones	5621	4619
4	Debris	4474	4588
5	Defects	13,295	12,056
6	Door	707	561
7	Floor	4751	4305
8	Human	14,318	13,358
9	Misc	597	236
10	Tape	6014	5604
11	Trolley	4811	4236
12	Clutter	659	538
13	Foam	174	51
14	Sawdust	253	136
15	Shard	39	109
16	Cellophane	372	395
17	Screws	169	157
18	Water	211	93
19	Obj. on Robot	12,551	11,716
20	Obj. on Robot 2	14,018	11,641

**Fig. 1:**
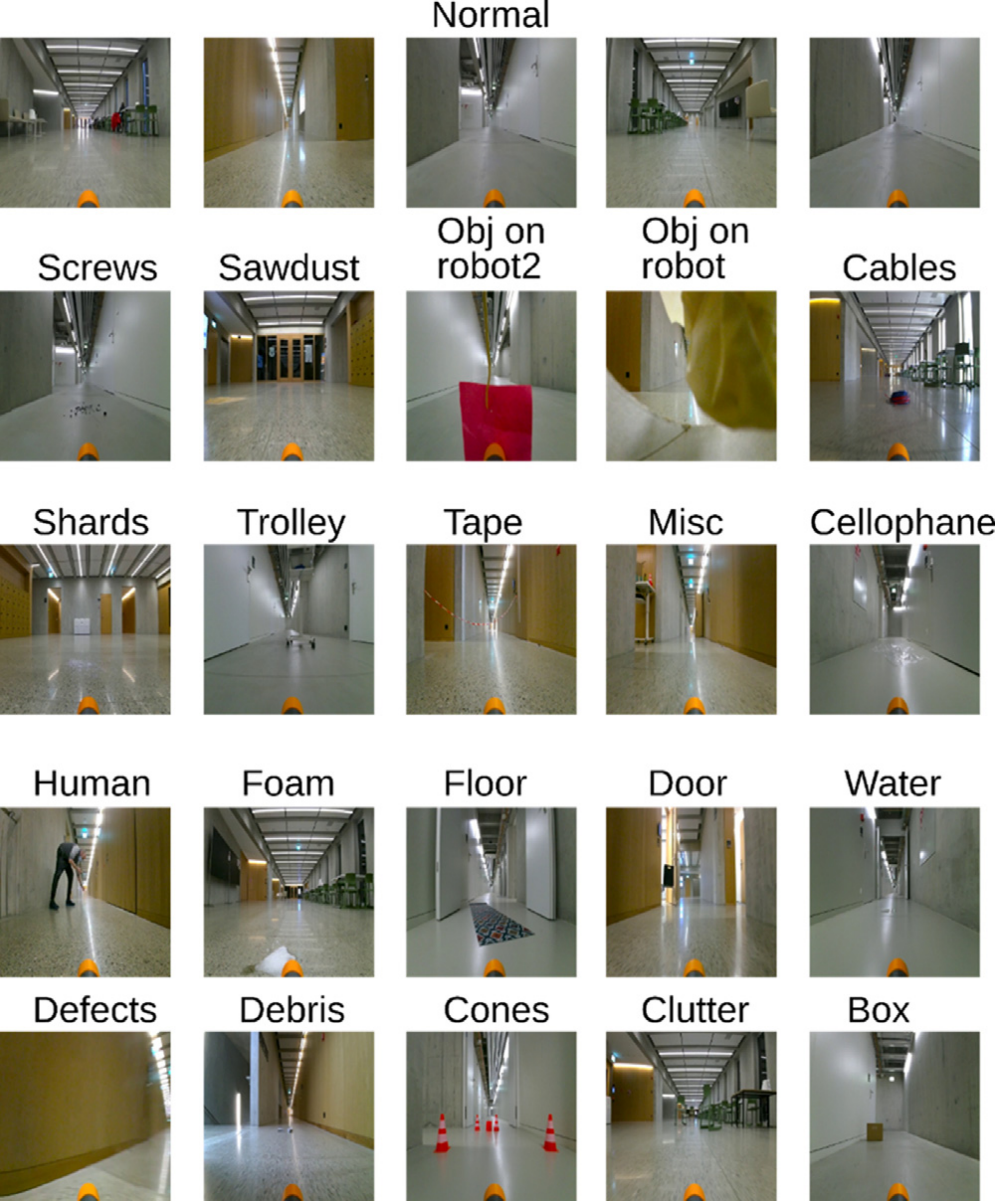
Samples of all anomalies and 5 samples of normal frames. (For interpretation of the references to color in this figure legend, the reader is referred to the web version of this article.)

In Fig. 1 we show an example for each class of anomaly and some examples of normal data in the different corridors.

## 3 Experimental Design, Materials and Methods

### 3.1 Data Collection

We used a DJI Robomaster S1, a wheeled ground robot; the robot provides a 120˚ field of view camera with a maximum resolution of 2560 × 1440. The camera, capable of recording full-HD videos at 30fps, is mounted on a gimbal. In addition, the robot is fitted with omnidirectional wheels and combined with the gimbal, it allows for a decoupling of the direction of movement from camera's pointing direction. This was exploited, for example, to simulate problems with the robot; frames acquired in this condition are labeled as *defects*.

The data collection was structured in multiple phases, all with the same setup, in order to capture the environments in different moments.

Each phase consisted in recordings of multiple sorties; for each recording we keep track of its length, corridor location, presence of anomalies, illumination, and direction of traversal. In all videos the robot moves at the center and along the length of the corridor.

For some classes of anomalies that were easier to replicate, we recorded multiple times the same combination of location and sortie duration but with different illumination, starting point and anomaly placement.

For short anomalous videos we recorded at least 5 sorties in each location and for long videos two sorties, one for each direction of traversal.

We record more than 550 sorties to cover as much environment variations as possible.

All videos are recorded to contain either only normal samples or only anomalous samples.

Regardless of the sortie characteristic, all recordings follow the same pattern:

- first, select a corridor and a direction of traversal,
- if needed, anomalies are placed in the corridor manually,
- then, the robot is placed at a starting point (the start of the corridor for long video, a random place for short ones but at max 2m from the anomaly, if present),
- finally, the recording is started (either on board on a microSD or on a smartphone via the Robomaster app) and the robot is teleoperated via the smartphone app.

**Fig. 2:**
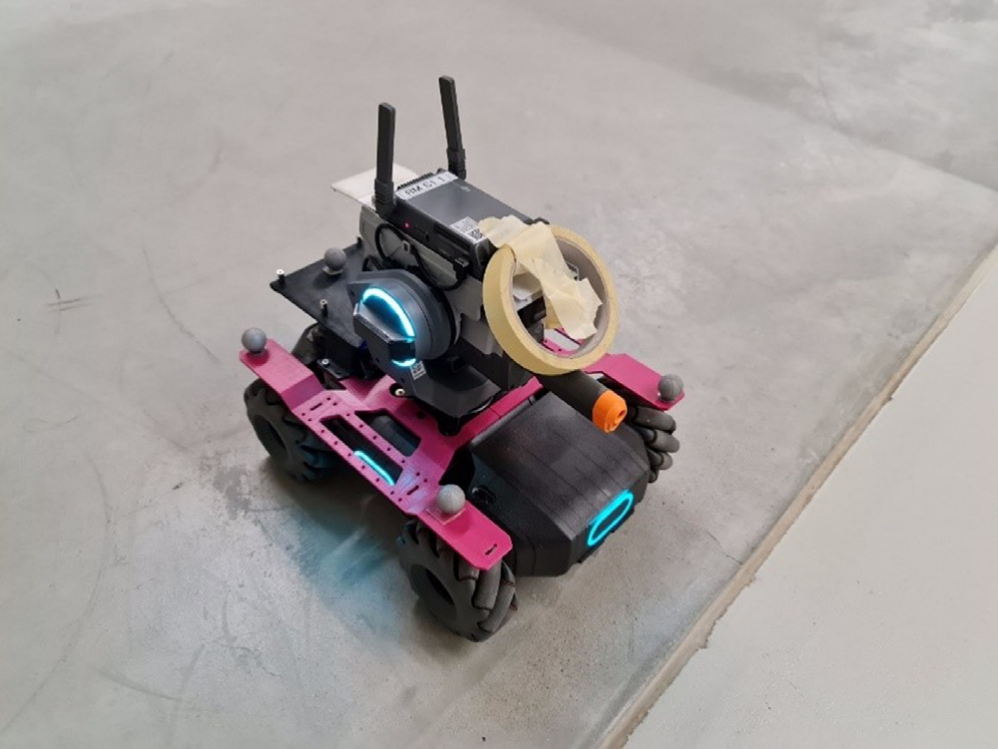
Example of an anomaly fitted on the robot. (For interpretation of the references to color in this figure legend, the reader is referred to the web version of this article.)

**Fig. 3:**
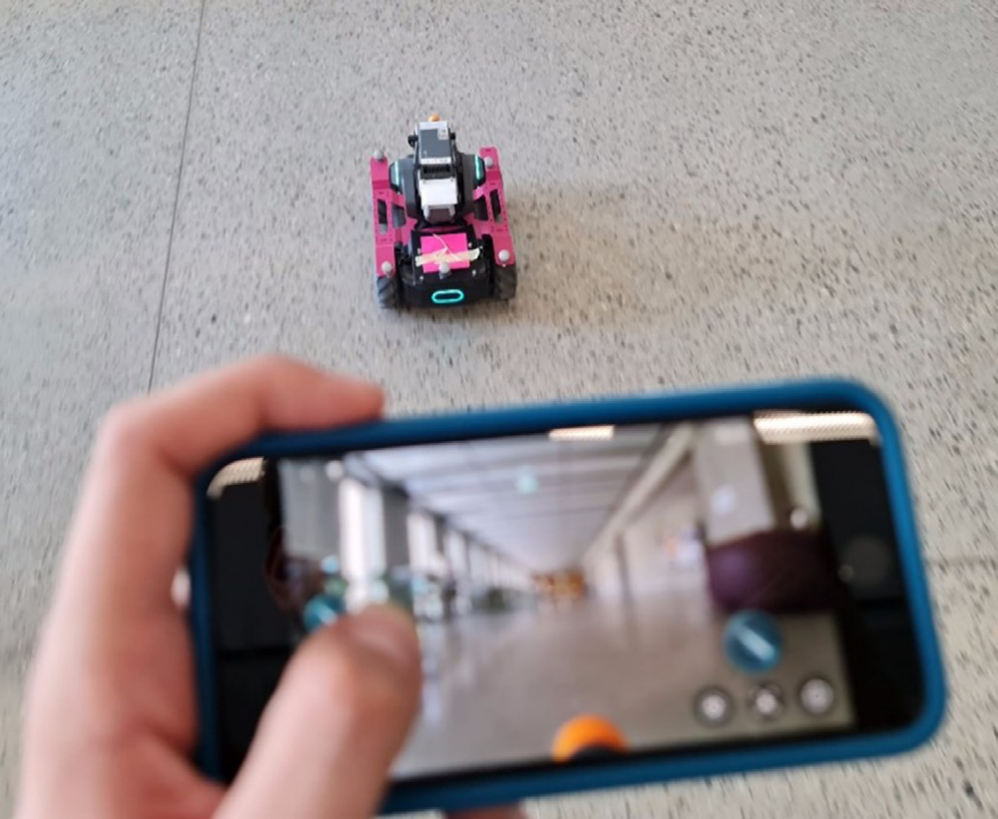
How the robot was controlled during data collection. (For interpretation of the references to color in this figure legend, the reader is referred to the web version of this article.)

See Fig. 2, Fig. 3 to see examples of data collection.

We tested other means of teleoperation, namely via laptop using ROS or via a controller connected to the robot, but the smartphone app was the most reliable and consistent method of teleoperation.

We catalogued all the videos with the relevant information and the result is a list of unique video recordings with a resolution of 1280 × 720 at 30 fps.

### 3.2 Data Processing

Since all videos are catalogued, we easily created the sets splits so that they are not overlapping and are balanced with respect to anomalies presence in the sets.

We did this programmatically using a custom Python script.

After assigning the video to the sets, all frames are extracted.

These frames are then renamed - using the naming convention described before - and are anisotropically resized to the resolution of 512 × 512 using OpenCV; the scripts used to extract and resize the frames are available in the *code.zip* folder.

The results are place in the sets folders and are compressed.

### 3.3 Feature Extraction

After frame creation, processing and splitting, we create the feature embeddings.

Arbitrarily, we decided to use CLIP ViT-B/32 and with a custom Python script based on the example in the CLIP repository [11] README; we produce a 512-sized embeddings vector for each frame.

These vectors, represented as PyTorch tensors, are stacked for efficiency in a 2D matrix. Each one of these matrices represent a set and is then saved in one of the four .pt (or .npy) files in *embeddings*; this is done using a basic PyTorch Python script that saves the extracted embeddings.

## Ethics Statements

This study does not involve experiments on humans or animals subjects. The data contains non-recognizable images of some people; their consent was obtained.

## CRediT authorship contribution statement

**Dario Mantegazza:** Conceptualization, Methodology, Software, Validation, Formal analysis, Investigation, Resources, Data curation, Writing – original draft, Supervision. **Alind Xhyra:** Methodology, Software, Validation, Formal analysis, Investigation. **Luca M. Gambardella:** Funding acquisition, Project administration. **Alessandro Giusti:** Funding acquisition, Project administration, Supervision, Conceptualization, Resources, Writing – review & editing. **Jérôme Guzzi:** Funding acquisition, Project administration, Supervision, Resources, Conceptualization, Writing – review & editing.

## Declaration of Competing Interest

The authors declare that they have no known competing financial interests or personal relationships that could have appeared to influence the work reported in this paper.

## Data Availability

Hazards&Robots: A Dataset for Visual Anomaly Detection in Robotics (Original data) (Zenodo).
